# The Use of Amnion-Derived Cellular Cytokine Solution (ACCS) in Accelerating Closure of Interstices in Explanted Meshed Human Skin Grafts

**Published:** 2009-03-25

**Authors:** M. Georgina Uberti, Francis Ko, Yvonne N. Pierpont, Erika L. Johnson, Terry E. Wright, Charlotte A. Smith, Martin C. Robson, Wyatt G. Payne

**Affiliations:** ^a^ Institute for Tissue Regeneration, Repair, and Rehabilitation, Bay Pines VAHCS, Bay Pines, FL; ^b^ Division of Plastic Surgery, Department of Surgery, University of South Florida, Tampa, FL; ^c^ Stemnion, Inc, Pittsburgh, PA

## Abstract

Meshed, split-thickness skin grafts, especially when required to be widely spread, do not obtain immediate biologic closure. In patients with burns that cover a large percentage of the body surface area, this leaves the patient at risk for metabolic problems and life-threatening infection. **Objective:** The purpose of this study was to determine whether amnion-derived cellular cytokine solution could improve epithelialization kinetics and accelerate closure of meshed skin graft interstices. **Methods:** Human meshed, split-thickness skin grafts were explanted to athymic “nude” rats and treated with 3 different regimens of amnion-derived cellular cytokine solution (groups I, II, and III) or normal saline (group IV) as a control. Serial wound tracings of unepithelialized interstitial wound areas were compared over time. Two different preparations of amnion-derived cellular cytokine solution were also compared with one another, one containing animal components and the other free of animal components. **Results:** Only 67.03% of interstices in control animals closed by day 9. This compared with 92.2% closure for group I, 83.72% for group II, and 90.64% for group III. Interstices in all 3 groups treated with amnion-derived cellular cytokine solution (with or without animal-derived components) closed faster statistically than in the control animals (*P* < .05). There were no statistical differences among the 3 amnion-derived cellular cytokine solution–treated groups. **Conclusions:** These data suggest that epithelialization kinetics and interstitial closure of meshed skin grafts can be accelerated with the use of amnion-derived cellular cytokine solution, a physiologic cocktail of cytokines, and provide support for a future clinical trial.

Amnion-derived multipotent progenitor (AMP) cells serve as the basis for a platform technology being developed for the treatment of burn wounds.^1^,^2^ These cells are isolated from the noncontroversial, full-term placenta and are unlike most stem cells in that they have been demonstrated to be nontumorigenic and nonimmunogenic.^3^ Amnion-derived cellular cytokine solution (ACCS), a secreted product of AMP cells, is a cocktail of cytokines existing at physiologic levels.^4^ It is being developed to treat 4 types of burn wounds: the partial-thickness burn, which, if protected, can heal by epithelialization; the excised full-thickness burn, which will eventually have to be closed; the interstitial wounds seen in meshed skin grafts; and the skin graft donor site. Meshed, split-thickness skin grafts, especially when required to be widely spread, do not obtain immediate biologic wound closure.^5^ In patients with burns that cover a large percentage of total body surface area, the remaining open wounds in the interstices of the mesh leave the patient at risk for metabolic problems and life-threatening infection. Smith et al^5^ showed in a model of explanted human meshed skin grafts onto an athymic “nude” rat that individual growth factors such as KGF-2, bFGF, and TGF-β_2_ could accelerate epithelialization kinetics and interstitial wound closure. The purpose of this study was to evaluate whether a physiologic cocktail of cytokines, ACCS, accelerates interstitial wound closure in human meshed skin grafts.

## MATERIALS AND METHODS

An animal model of human meshed, split-thickness skin grafts explanted onto athymic “nude” rats was used to evaluate ACCS as a cocktail of physiologic cytokines.^5^–^7^ This model allows for the accurate measurement of closure of graft interstices serially over time. The ACCS was prepared for the experiment as follows: The AMP cells were grown to confluency in 1 of 2 serum-free media. The first contained animal-derived components and the second contained no animal-derived components. Once the cells had grown to confluency, using proprietary techniques, the supernatant was harvested as ACCS.

Same-day, birth-dated, congenitally outbred, athymic “nude” rats (Harlan Sprague Dawley Inc, Indianapolis, Ind) weighing 250 to 300 g were used. Because of their immunocompromised state, the animals were housed in a pathogen-free barrier facility and individually caged in microisolators that were kept in a laminar-flow room.^5^–^7^ All animal supplies, such as food, water, and bedding, were sterilized by autoclave before use. The rats received food and water ad libitum. Sterile gloves, gowns, caps, and shoe covers were worn at all times by personnel who were handling the animals. All surgery was performed with the use of strict aseptic techniques and under laminar-flow hoods. All experiments followed a protocol approved by the Bay Pines VAHCS Animal Care Use Committee and complied with the National Research Council's criteria for humane care of animals.

Twenty male, athymic “nude” rats were divided into 4 equal groups of 5 animals each (120 interstitial wounds per animal or 600 wounds per group) to have their skin grafts treated with ACCS (free of animal-derived components) at 0.01 mL/cm^2^ daily (group I) or on alternate days (group II); original ACCS (with animal-derived components) at 0.01 mL/cm^2^ daily (group III), or with 0.9% NaCl daily (group IV) as a control. Fresh split-thickness human skin was meshed 1:1.5 with the use of aseptic techniques. Contracts with a commercially available, certified tissue bank specified that the skin used be harvested from a single site on a single cadaveric donor with an electric dermatome. The skin was 0.014- to 0.020-in thick. Skin sample cultures and donor serology confirmed that the tissue was negative for pathogenic organisms, hepatitis B and C viruses, and HIV. Cell viability immediately before explantation was confirmed to be more than 90% according to the Trypan Blue exclusion test.

**Figure 1:**
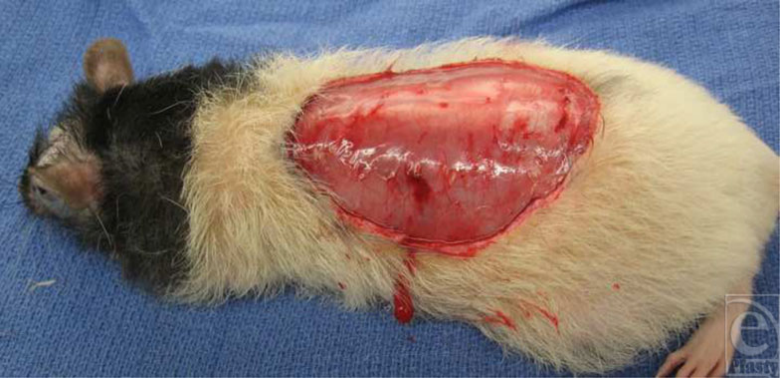
A 30-cm^2^ defect on the dorsum of a “nude” rat.

**Figure 2:**
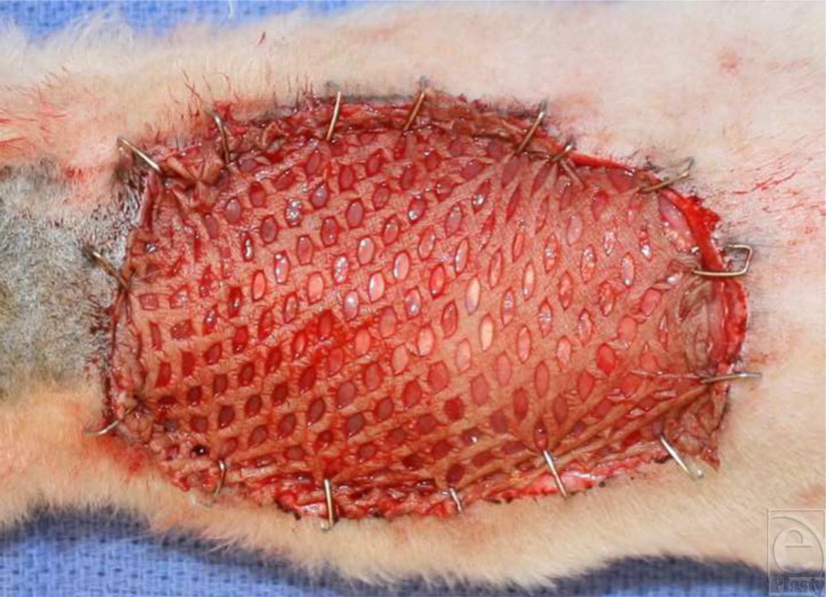
A maximally spread, meshed, split-thickness skin graft on a “nude” rat.

With the use of strict aseptic techniques and working within a unidirectional airflow biologic hood, the rats were anesthetized with intraperitoneal pentobarbital (Nembutal) at a dose of 35 mg/kg body weight. After the dorsal skin was treated with povidone-iodine solution, a standardized full-thickness area (30-cm^2^) of skin and panniculus carnosa were excised (Fig 1). The meshed skin graft was pinned to a corkboard that had a cutout area the same size as the wound that was inflicted to each animal.^6^,^7^ The graft was then spread to the greatest degree possible in an effort to maximize the extent of graft interstices. This maximally spread meshed graft was then grafted to the wound on the animal and fixed with staples to the surrounding wound edges (Fig 2). The grafts were treated with the test solutions by pumping the solutions from a pump actuator at a rate of 0.01 mL/cm^2^ of graft. The grafts were dressed with N-Terface nonadherent gauze (Winfield Laboratories, Inc, Richardson, Tex) and covered with an 8-ply gauze dressing.

**Figure 3:**
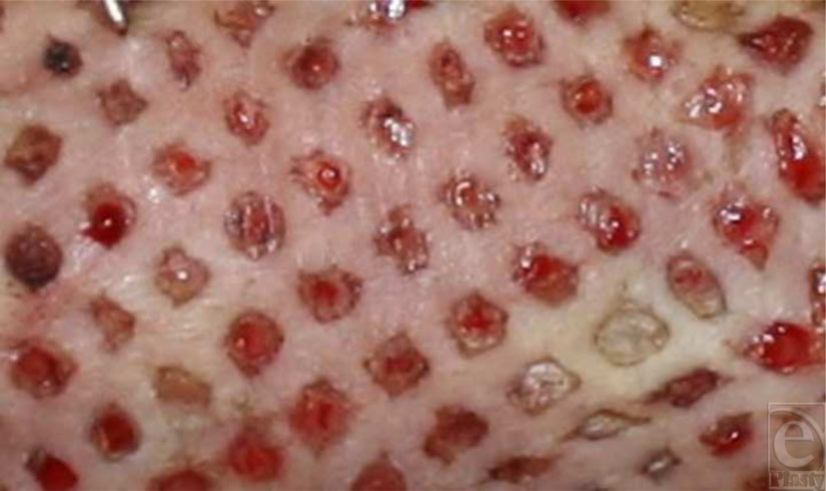
The photograph of a “nude” rat in group II on day 6 demonstrating complete epithelialization of some interstices and partial epithelialization of other interstices.

Standardized photography of the grafts was performed before the initial dressing and daily thereafter (Fig 3). The dressing was changed at the time that the daily photograph was taken. A digital, single-lens reflex camera with a macro lens was mounted onto a camera stand. A centimeter ruler was included in each photograph. The photographs were analyzed and an arbitrarily sized designated analysis area was chosen in the central area of each graft. With the use of planimetry software, the grafts were analyzed as had been done in previous studies.^5^–^7^ The interstitial areas that remain open within the designated analysis area of each graft were measured. Neoepithelial areas were calculated by subtracting daily measurements of interstitial areas from the original measurements of those interstitial areas. A percentage of neoepithelialization was then determined for designated analysis area by the following equation:

Percentage Neoepithelialization = (Neoepithelial Area/Designated Analysis Area) × 100

Data from each day were compared among the 4 groups by 1-way analysis of variance and between pairs of groups by the Fisher least significance difference test with an α value of .05. Histologic sections were obtained through the meshed graft interstices on day 9 to demonstrate that the interstices have closed by the process of epithelialization rather than contraction.

## RESULTS

**Figure 4:**
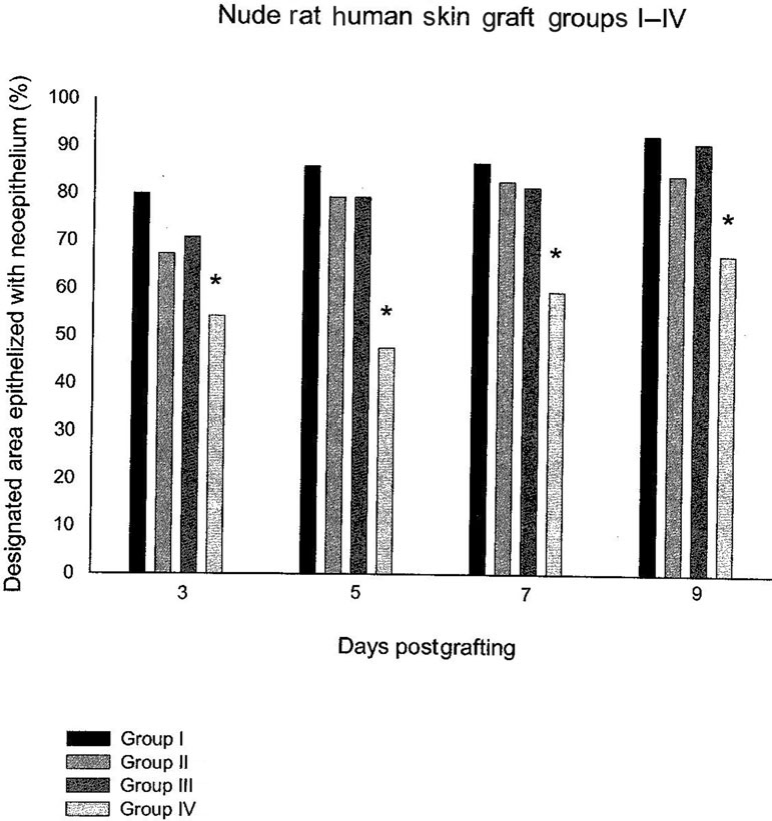
Epithelialization rates of 600 wounds per group: group I, amnion-derived cellular cytokine solution (ACCS) without animal-derived components, daily treatment; group II, ACCS without animal-derived components, every other day treatment; group III, ACCS containing animal-derived components, daily treatment; and group IV, saline daily. *P < .05.

Seventeen of the animals achieved variable closure of interstices by epithelialization. The dressings were slightly adherent in 3 animals so as to possibly disrupt the new epithelium. Therefore, these 3 animals were eliminated from statistical evaluation (1 animal each from groups I, III, and IV). Because the epithelialization rate was leveling off by day 9, the animals were humanely euthanized on day 10. Only 67.03% of the interstices in control animals closed by day 9 (Fig 4). This compared with 92.2% closure for group I, 83.72% for group II, and 90.64% for group III. Interstices in all 3 groups treated with ACCS (with or without animal-derived components) closed faster statistically than in the control animals (*P* < .05) (Fig 4). There were no statistical differences among the 3 ACCS-treated groups. The ACCS-treated group, using ACCS prepared without any animal-derived components and applied to the grafts daily, had the greatest degree of epithelial closure of interstices (group I), although the difference was not statistically significant. The model showed comparability between ACCS prepared with media containing animal-derived components (group III) and ACCS prepared without animal-derived components (groups I and II). Histology of sections from groups I to III were identical, demonstrating that normal epithelialization occurred in all healed grafts and that interstices closed by epithelialization and not by contracture.

## DISCUSSION

Early excision and wound closure have significantly decreased mortality and morbidity of patients with burns. Available donor sites for autologous skin grafts decrease as the total size of the burn wound in need of excision increases. Meshing of the skin grafts allows an increase in the area a given graft can cover. However, the open area of the meshed graft interstices is similar to all open wounds; it increases the metabolic demand on the patients and they remain prone to life-threatening invasive infection.^5^,^7^ Because several single growth factors have been shown to improve epithelialization kinetics and accelerate interstitial wound closure in mesh grafts,^5^ it was hypothesized that a cocktail of growth factors would be similarly effective.

Normal wound healing is accomplished by a combination of cytokines, and they occur in a “natural” cascade.^8^ Stem cells and stem cell¼like multipotent cells are known to produce cytokine growth factors that serve as mediators to the cellular processes of the wound healing scheme.^9^ Steed et al^4^ reported that AMP cells secreted a solution with 6 consistent cytokines in their cocktail. These 6 cytokines were PDGF, VEGF, angiogenin, TGF-β_2_, TIMP-1, and TIMP-2. The levels of the 6 naturally occurring cytokines in ACCS were compared with levels of those cytokines from normally healing wounds reported in the literature.^4^ ACCS was determined to contain 6 cytokines at physiologic levels. It was for this reason that ACCS was postulated to be effective in these wounds. The single growth factors reported in this model by Smith et al^5^ were at higher levels than the physiologic levels in ACCS. Also, ACCS has TIMP-1 and TIMP-2 in it, which can inhibit matrix metalloproteinases produced in the open granulating wounds.^10^ The first ACCS harvested was from AMP cells grown in a medium containing animal-derived components. Following success with several proof-of-concept experimental wound healing models, AMP cells were grown in a medium free of animal-derived components, as would be required for future clinical trials. Therefore, ACCSs produced in both media were used in this study.

There was *no* observed toxicity among the animals. All survived the experiment. The dressings of 3 animals adhered to their wounds and these animals were, therefore, eliminated from statistical evaluation because the rate of epithelialization might have been affected. The dose was 0.01 mL/cm^2^ for each application of ACCS or normal saline. Groups I, III, and IV had the application daily and group II had the application every other day. Although the daily application of ACCS developed in either medium allowed faster epithelialization than the every other day application, these differences were not statistically significant (Fig 4).

It is clear that ACCS improved epithelialization kinetics and accelerated wound closure in this model. Both ACCS preparations were effective. The importance of the ACCS without animal components is that the Food and Drug Administration desires that biologic products do not contain animal components when used in clinical trials. Because human meshed skin grafts were used with ACCS from a human source, there is reason to believe that the results may be extrapolated to the human situation. Small animal experiments will not allow definitive dose-response data, which will have to be determined during human clinical trials. Therefore, the next step in confirming the efficacy of this treatment would be to perform a prospective, randomized, blinded, placebo-controlled clinical trial that would test the ability of ACCS to accelerate the interstitial closure of meshed skin grafts in patients with burns.
